# Case Report: Leathery black plaque on the temple and scalp

**DOI:** 10.12688/f1000research.142548.1

**Published:** 2023-11-27

**Authors:** Kaveri Rusia, Bhushan Madke, Soham Meghe, Yash Kashikar

**Affiliations:** 1 Department of Dermatology, Datta Meghe Institute of Higher Education and Research, Wardha, Maharashtra, 442001, India

**Keywords:** hamartoma, nevus sebaceous, scalp, case report

## Abstract

**Background:**

Epidermal nevus sebaceous, commonly known as the nevus sebaceous of Jadassohn, is a congenital sebaceous hamartoma. It typically manifests as a single yellowish plaque across the head and neck and is composed of sebaceous glands. It commonly occurs during infancy and grows during puberty. Usually, it follows a benign course; however, in a few cases, it can be malignant. This is the case of a 13-year-old child with verrucous plaques on the temple and scalp.

**Case report:**

We report the case of a 13-year-old boy with a steadily developing hyperpigmented verrucous plaque on the scalp and ipsilateral side of his face. A dermoscopic examination revealed ridges and fissures in a cerebriform pattern with yellowish-gray globules and a papillary appearance. Physical examination and laboratory tests revealed no abnormalities. Biopsies were taken from the scalp and temple area, and the findings were consistent with the diagnosis of nevus sebaceous. The patient was referred to a plastic surgeon for a staged excision.

**Conclusions:**

We describe a unique example of a sebaceous nevus that affected the scalp and ipsilateral side of the face. As this hamartomatous growth carries the risk of cancer development, a dermatologist must identify the condition and begin treatment before malignant transformation occurs. This example of multiple verrucous plaques is an exception.

## Introduction

Nevus sebaceous (NS), initially described by Jadassohn, is a complicated hamartoma that typically develops on the face or scalp and has an epithelial or adnexal origin. ^1^

It can appear at birth or develop in infancy and increases during puberty, suggesting a hormonal influence. It can occasionally be found in other locations, such as the trunk or the oral or vaginal mucosa, although it mostly affects the scalp. Less frequently, it affects the preauricular area and neck. ^2^

Nevus sebaceous of Jadassohn (NSJ) develops in three stages. It manifests as isolated, well-circumscribed, smooth, yellowish plaques without hair during the infantile period. It becomes more noticeable with a verrucous or mamillated appearance during puberty. The last stage is characterised by peripheral telangiectasias and a nodular or tumoral appearance. ^3^

Many neoplasms develop alongside NS as proliferative growth begins. Both benign and malignant tumors have been reported to grow in NS. NS can be a site of basal cell cancer, syringocystadenoma papilliferum, trichoblastoma, and hidradenoma. ^4^

## Case report

A 13-year-old boy visited the dermatology outpatient department on 8 ^th^ September 2023 with a raised lesion on his scalp since birth and a lesion that had spread to the left side of the face over ten years. The ophthalmological, neurological, or cutaneous systems did not exhibit any abnormalities during physical examination. These skin lesions had not previously occurred in the family. The results of all laboratory tests, including the kidney function test, liver function test, urine examination, and complete blood count were within normal ranges. The patient had no other complaints.

**Figure 1.**
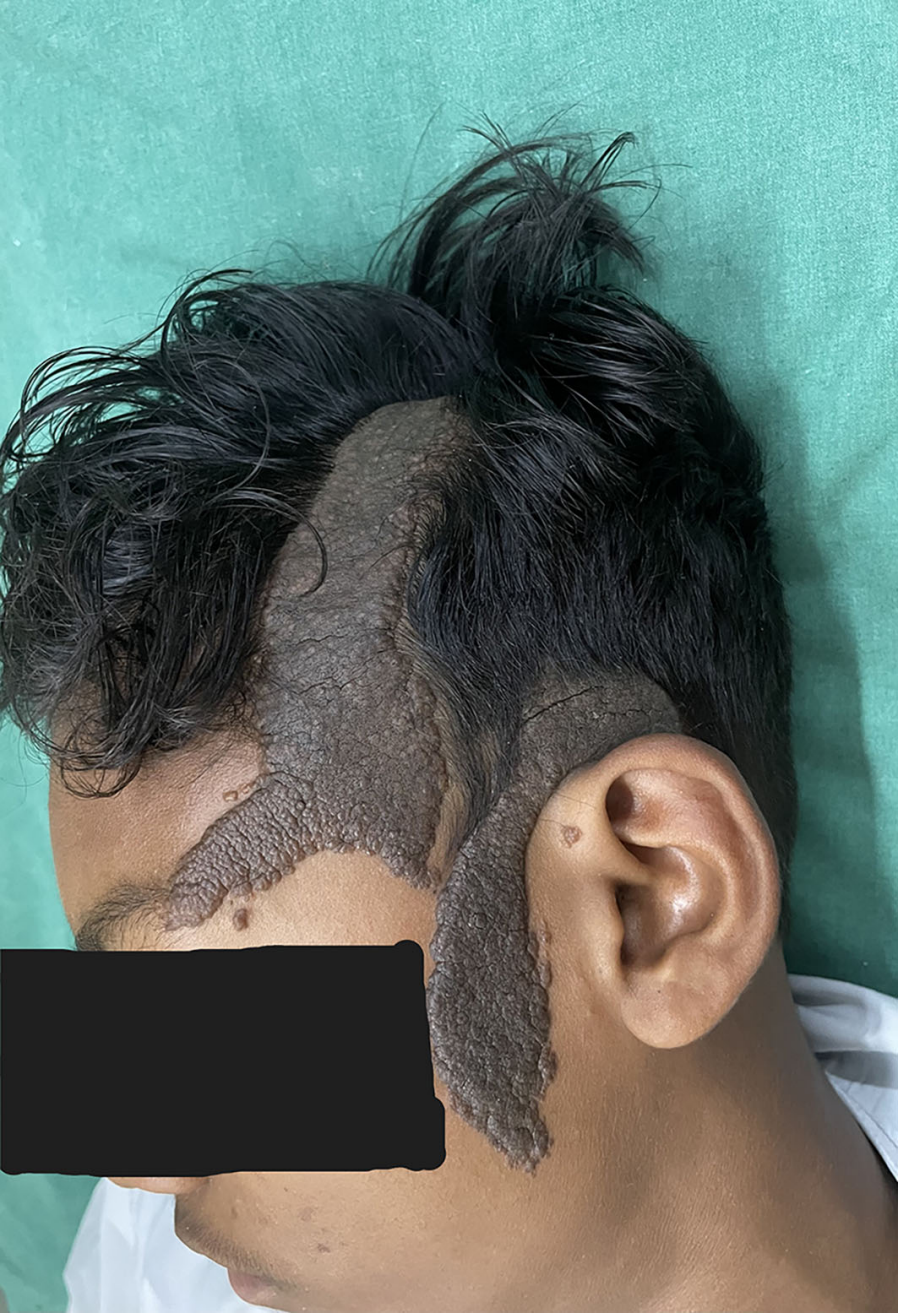
Verrucous plaque on frontal, temporal and preauricular area. (Written informed consent for publication of their clinical details and clinical images was obtained from the relatives of the patient).

**Figure 2.**
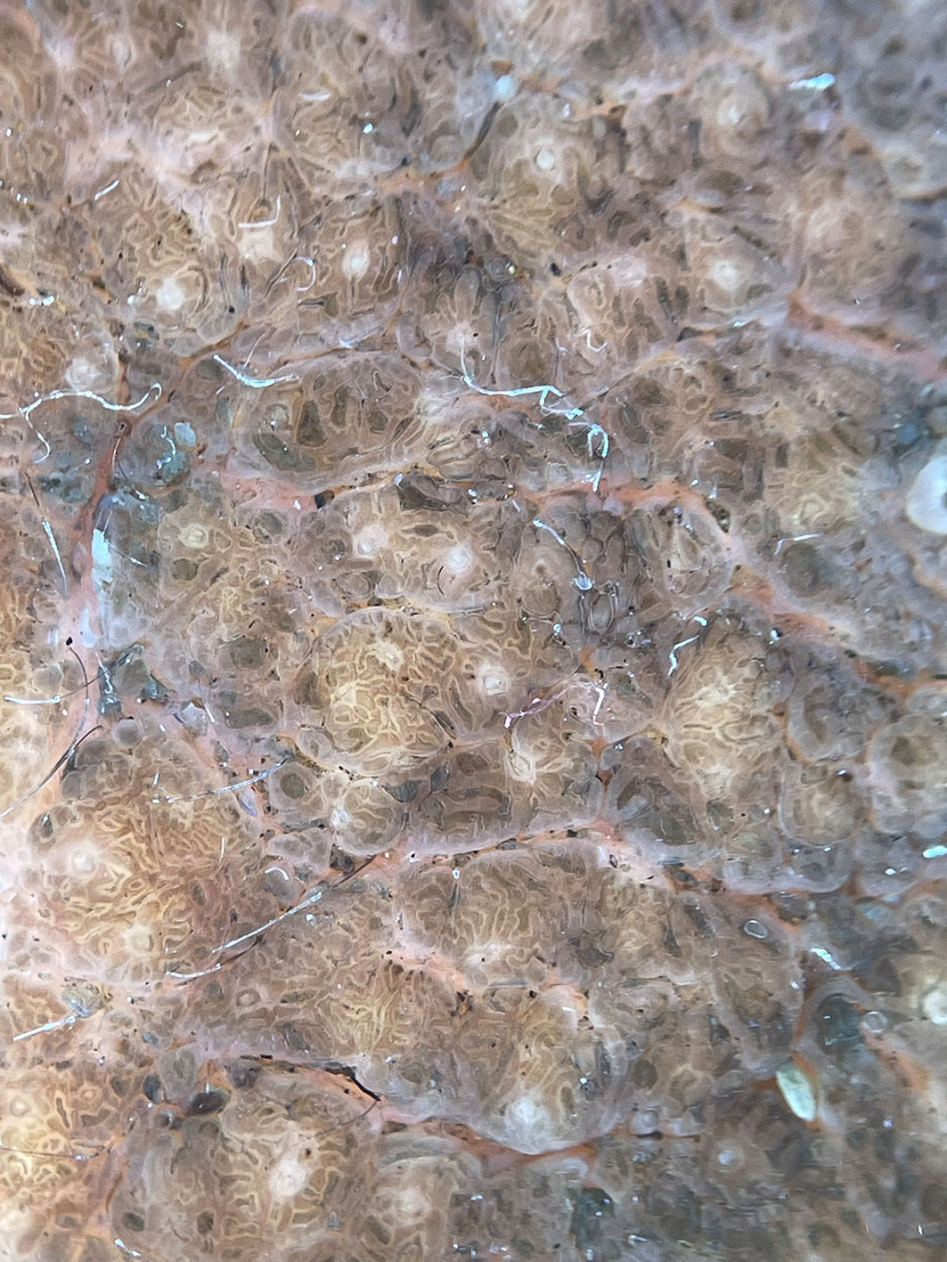
Ridges and fissures in a cerebriform pattern with yellowish grey globules and papillary appearance.

**Figure 3.**
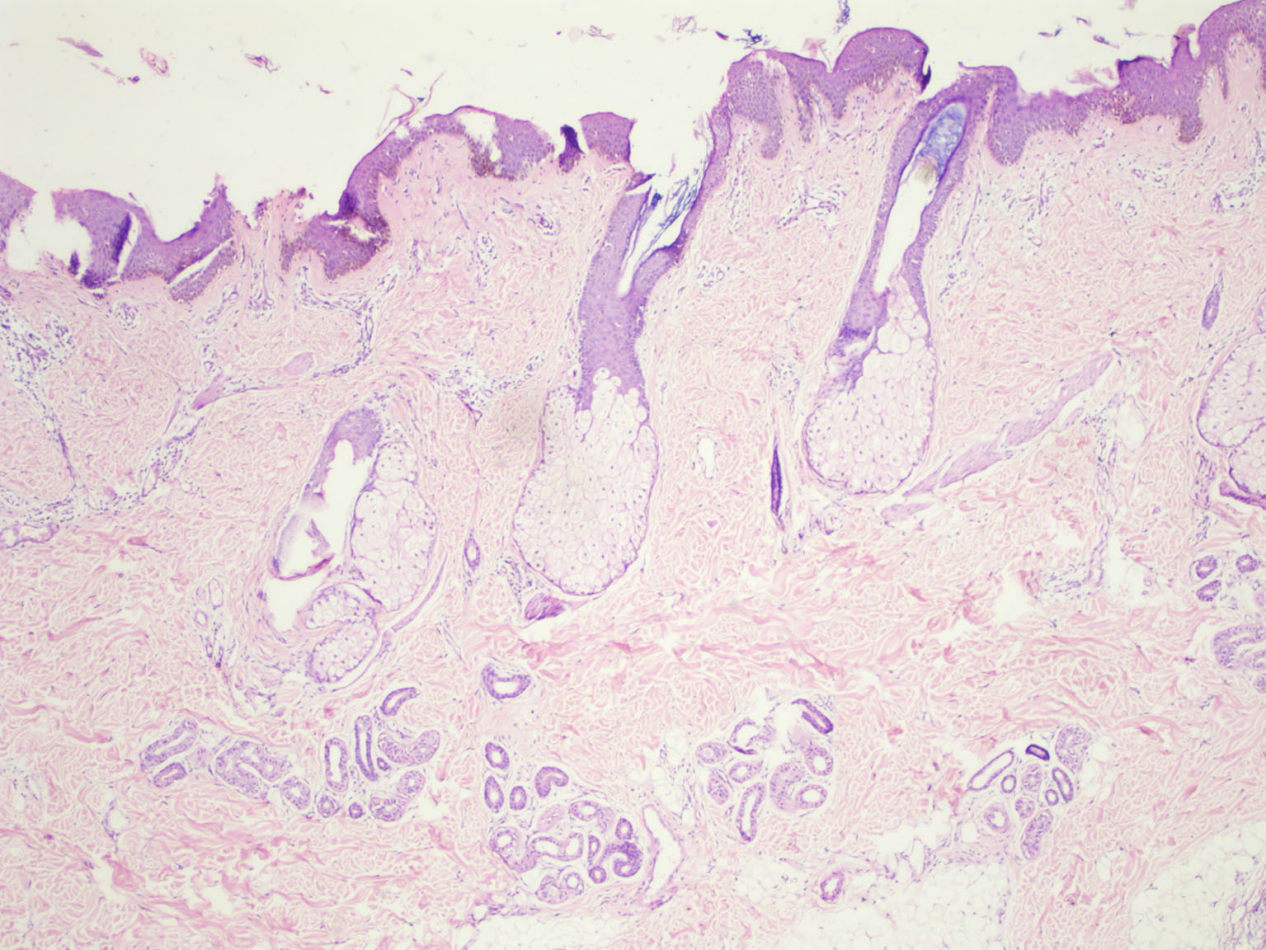
There are immature and mature sebaceous gland with sebaceous hyperplasia along with primitive hair follicles with acanthosis, acanthosis, papillomatosis and mild hyperkeratosis of epidermis.

On cutaneous examination, a well-demarcated hyperpigmented verrucous plaque with a size of 8 × 4 cm was present on the frontal area of the scalp extending down to involve the forehead and a 7 × 3 cm plaque was present on the temporoparietal area and left preauricular area [ Figure 1]. Based on the patient’s medical history and physical examination, the possible differential diagnoses were identified as congenital melanocytic nevus, giant seborrhoeic keratoses, and verrucous epidermal nevus. However, a thorough examination through dermoscopy and histology conclusively ruled out these possibilities. On dermoscopic examination, ridges and fissures were present in a cerebriform pattern with yellowish-grey globules and a papillary appearance [ Figure 2]. Histopathological examination revealed acanthosis, papillomatosis, and mild hyperkeratosis. There were immature and mature sebaceous glands with sebaceous hyperplasia and primitive hair follicles [ Figure 3]. The diagnosis of nevus sebaceous was established based on clinical presentation, dermoscopic findings, and histological analysis. The patient was referred to a plastic surgeon on 8 ^th^ September 2023 for a staged surgical excision of the nevus sebaceous. Our dermatology department does not offer plastic surgery services, hence the referral. Unfortunately, the patient was lost to follow-up after the referral, and we do not have any further information available.

## Discussion

Nevus sebaceous is a condition that appears at birth and increases in size with age. The exact cause of this condition is still uncertain, but recent studies have shown that it may be linked to women who have tested positive for the human papillomavirus or carry mutations in the PTCH gene. ^5^^,^^6^

Nevus sebaceous can present as one of the manifestations of Epidermal Nevus Syndrome. ^7^ There are some hereditary syndromes, including didymosis aplasticosebacea and SCALP (sebaceous nevus, central nervous system malformations, aplasia cutis congenita, limbal dermoid, and pigmented nevus) syndrome, that may present nevus sebaceous as a symptom. This condition typically appears as a smooth, yellowish-orange, round, oval, or linear plaque, mostly on the scalp, leading to alopecia. ^5^

A previous study found that nevus sebaceous can occur in multiple locations, similar to verrucous epidermal nevi. ^8^ Nevus sebaceous is rarely reported in the literature to affect the scalp and ipsilateral side of the face. ^9^ In our case, the scalp and the ipsilateral side of the face were affected.

Several discussions have taken place regarding the emergence of secondary benign and malignant tumors inside the nevus sebaceous. While basal cell carcinoma development has been documented by multiple authors in adults, recent reports have also identified atypical malignant neoplasms such as eccrine porocarcinoma, sebaceous carcinoma, apocrine carcinoma, and squamous cell carcinoma developing inside the NS. ^10^^,^^11^

Although the timing of resection for nevus sebaceous therapy is debatable, most researchers feel that surgical excision is the preferred course of action. However, surgical excision to remove nevus sebaceous creates a linear scar. There are various therapeutic options, such as CO _2_ laser therapy, to reduce scarring. However, CO _2_ laser vaporization completely eradicates the sebaceous section of the nevus, which is located in the epidermis or papillary dermis. ^12^

## Conclusions

The primary take-away lesson from our case is as follows: We describe a unique example of a sebaceous nevus that affected the scalp and ipsilateral side of the face. As this hamartomatous growth carries the risk of cancer development, a dermatologist must identify the condition and begin treatment before malignant transformation occurs. This example of multiple verrucous plaques is an exception.

## Consent

Written informed consent for publication of their clinical details and clinical images was obtained from the relatives of the patient.

## Data Availability

All data underlying the results are available as part of the article and no additional source data are required.

## References

[1] Lin HC Lee JY Shieh SJ : Large, papillomatous, and pedunculated nevus sebaceous. *J. Dermatol.* 2011 Feb;38(2):200–202. 10.1111/j.1346-8138.2010.00957.x 21269322

[2] Kelati A Baybay H Gallouj S : Dermoscopic analysis of nevus sebaceus of Jadassohn: a study of 13 cases. *Skin Appendage Disord.* 2017 May 2;3(2):83–91. 10.1159/000460258 28560218 PMC5436057

[3] Sahu P Lakra S Dayal S : Nevus sebaceous on face: Histopathological and dermoscopic correlation. *Indian Dermatol. Online J.* 2020 Sep;11(5):878. 10.4103/idoj.IDOJ_113_19 33235879 PMC7678547

[4] Ankad BS Beergouder SL Domble V : Trichoscopy: the best auxiliary tool in the evaluation of nevus sebaceous. *Int. J. Trichology.* 2016 Jan;8(1):5–10. 10.4103/0974-7753.179394 27127368 PMC4830180

[5] Moody MN Landau JM Goldberg LH : Nevus sebaceous revisited. *Pediatr. Dermatol.* 2012 Jan;29(1):15–23. 10.1111/j.1525-1470.2011.01562.x 21995782

[6] Carlson JA Cribier B Nuovo G : Epidermodysplasia verruciformis–associated and genital-mucosal high-risk human papillomavirus DNA are prevalent in nevus sebaceus of Jadassohn. *J. Am. Acad. Dermatol.* 2008 Aug 1;59(2):279–294. 10.1016/j.jaad.2008.03.020 18638629

[7] Happle R : The group of epidermal nevus syndromes: Part I. Well defined phenotypes. *J. Am. Acad. Dermatol.* 2010 Jul 1;63(1):1–22. 10.1016/j.jaad.2010.01.017 20542174

[8] Cribier B Scrivener Y Grosshans E : Tumors arising in nevus sebaceus: a study of 596 cases. *J. Am. Acad. Dermatol.* 2000 Feb 1;42(2):263–268. 10.1016/S0190-9622(00)90136-1 10642683

[9] Chi SG Kim JY Kim HY : Multiple nevus sebaceous occurring on the scalp and on the contralateral side of the face. *Ann. Dermatol.* 2011 Aug 1;23(3):389–391. 10.5021/ad.2011.23.3.389 21909216 PMC3162275

[10] Correale D Ringpfeil F Rogers M : Large, papillomatous, pedunculated nevus sebaceus: a new phenotype. *Pediatr. Dermatol.* 2008 May;25(3):355–358. 10.1111/j.1525-1470.2008.00682.x 18577043

[11] Jadassohn J : Bemerkungen zur histology der systematisierten naevi und ubertigdrusen naevi. *Arch. Dermatol. Syphilol.* 1895;33:355–372. 10.1007/BF01842810

[12] Ashinoff R : Linear nevus sebaceus of Jadassohn treated with the carbon dioxide laser. *Pediatr. Dermatol.* 1993 Jun;10(2):189–191. 10.1111/j.1525-1470.1993.tb00053.x 8346119

