# Supplementary material for: Case Report: Leathery black plaque on the temple and scalp
Source: F1000Res. 2024 Apr 8;12:1514. Originally published 2023 Nov 27. [Version 2] doi: 10.12688/f1000research.142548.2 (PMC11066521; doi:10.12688/f1000research.142548.2)
Supplement: Peer review history

# Reviewer report: Reviewer response for version 2

**DOI:** 10.5256/f1000research.164539.r264025

**Published:** 2024-05-02

**Authors:** Aswath Rajan

**Affiliations:** 1 Goa Medical College, Goa, India

Well written, relevant corrections made.

Are enough details provided of any physical examination and diagnostic tests, treatment given and outcomes?

Partly

Is the case presented with sufficient detail to be useful for other practitioners?

Partly

Is sufficient discussion included of the importance of the findings and their relevance to future understanding of disease processes, diagnosis or treatment?

Partly

Is the background of the case’s history and progression described in sufficient detail?

Yes

Reviewer Expertise:

dermatology

I confirm that I have read this submission and believe that I have an appropriate level of expertise to confirm that it is of an acceptable scientific standard.

# Reviewer report: Reviewer response for version 1

**DOI:** 10.5256/f1000research.156109.r251438

**Published:** 2024-03-28

**Authors:** Aswath Rajan

**Affiliations:** 1 Goa Medical College, Goa, India

Article is very well written. However here are following few suggestions/queries that could add more value to it.

 I had copied your statement mentioned in **bold letters** and its queries below it. here as follows, 

**1. "As this hamartomatous growth carries the risk of cancer development, a dermatologist must identify the condition and begin treatment before malignant transformation occurs"**

**Q: a) **as the lesion is bigger, how did you confirmed that malignant transformation had not occurred in the lesion. what was the measures taken to identify malignancy change in the entire lesion.

      b) Was dermoscopy performed over the entire lesion! Was biopsy done on any suspicious part!

**​​​​​​2. "The results of all laboratory tests, including the kidney function test, liver function test, urine examination, and complete blood count were within normal ranges."**

**Q: **what was the relevance and what laboratory abnormalities  can be  possibly expected in such cases.

**3. "On dermoscopic examination, ridges and fissures were present in a cerebriform pattern with yellowish-grey globules and a papillary appearance"**

 Q: a) several dermoscopic features were missed out. Dermoscopy is the key factor in this case. As the biopsy cant be done over the entire lesion, dermoscopy can bridge the gap between clinical and histopathological findings.

 b) ridges and fissures seen in several other conditions like seborrheic keratosis, acanthosis nigricans, nevus. So What was the classical features in this case.

 c) mention about the clods/globules of different sizes and shapes

 d) several clods are black (indicates keratinous plug), bright black dots/globules indicates that it communicate with surface, whereas the dull black are intraepidermal one.

 e) was the biopsy performed on the same dermoscopic site.

 d) mention other close dermoscopic differential diagnosis and one or two points on its differentiation.

****

**4. "Several discussions have taken place regarding the emergence of secondary benign and malignant tumors inside the nevus sebaceous"**

**Q:** kindly mention for the readers,  what are the possible clinical features that alerts the malignant change and its relevant investigations for early detection of local and systemic invasion with  their likely management

**5." As this hamartomatous growth carries the risk of cancer development, a dermatologist must identify the condition and begin treatment before malignant transformation occurs".**

**Q:** Malignant transformation was repeatedly emphasized in the article. So Mention the role of latest non-invasive skin imaging technique such as high-frequency ultrasound, multispectral *imaging*, optical coherence tomography, reflectance confocal microscopy etc. also mention a word on molecular/genetic study if reported earlier for early diagnosis of malignancy.

Are enough details provided of any physical examination and diagnostic tests, treatment given and outcomes?

Partly

Is the case presented with sufficient detail to be useful for other practitioners?

Partly

Is sufficient discussion included of the importance of the findings and their relevance to future understanding of disease processes, diagnosis or treatment?

Partly

Is the background of the case’s history and progression described in sufficient detail?

Yes

Reviewer Expertise:

dermatology

I confirm that I have read this submission and believe that I have an appropriate level of expertise to confirm that it is of an acceptable scientific standard, however I have significant reservations, as outlined above.

# Response

**Published:** 2024-03-31

**Authors:** Kaveri Rusia

**Affiliations:** dermatology, datta meghe institute of higher education and research, wardha, maharashtra, India

Thank you for your comments.

 1)  We confirmed on clinical grounds that there were no clinical signs in the lesion to undergo a malignant transformation like ulceration, bleeding from the lesion or sudden increase in the size of the lesion. 

  A dermoscopy was performed on the entire lesion and in the manuscript we have shown the characteristic features. On clinical examination, there were no suspicious parts to undergo malignant transformation so a biopsy was performed from a random site.

 2) We performed the laboratory investigations since such cases may need surgical excision.

 3)  We have included the characteristic dermoscopic findings which were present in our case which is yellowish globules aggregated in clusters on a yellow background with cerebriform pattern of sulci and gyri.  In seborrheic keratosis, other than ridges and fissures multiple milia and comedone-like openings will be there. In acanthosis nigricans, there will be papillary projections with hyperpigmented dots and perifollicular pigmentation.

 4) The process of malignant degeneration is often accompanied by rapid morphological changes and other symptoms. Some of the morphological changes and symptoms include a change in the color of the skin, protruding mass, ulceration, change in size, and itching. Reflectance Confocal Microscopy can be performed to detect the malignant transformation.

 5) The non-invasive techniques like High-frequency Ultrasound are used to visualize skin and skin appendages for accurate depth and lateral border detection for skin malignant and benign tumors.

 The reflectance confocal microscopy allows in vivo evaluation of lesion of the microscopic extension of lesion and shows both anatomical features and individual cells.

 The presence of PTCH deletion, HRAS, KRAS mutation leads to malignant transformation in the nevus sebaceous.

# Reviewer report: Reviewer response for version 1

**DOI:** 10.5256/f1000research.156109.r241167

**Published:** 2024-02-23

**Authors:** Dipanjan Basu

**Affiliations:** 1 Pathology, University of Pittsburgh, Pittsburgh, Pennsylvania, USA

Nevus sebaceous of Jadassohn is known as a rare congenital malformation, grouped under hamartomas. Etiology is thought to be driven by somatic post-zygotic mutations in RAS genes among others. The clinical manifestation occurs as plaques and is commonly found on scalp and face, usually presenting with a verrucous appearance. The current study under review discusses a case of a 13-year-old child with verrucous plaques on the temple and scalp. The authors describe the case as Nevus sebaceous of Jadassohn based on clinical, dermoscopic and histological analysis. The case exhibits classic characteristics of a sebaceous nevus including hyperplasia of sebaceous glands.

 The incidence of nevus sebaceus is estimated approximately at 0.1% to 0.3% of all newborns without bias for sex or ethnicity. This case report is a valuable addition to previous reports to understand the nuances of this condition and would be a valuable source of knowledge for students and practicing clinicians. The authors described the background of the case’s history and progression in adequate detail including reference to current literature. However, the authors may include that RAS genes are also implicated in the development of this condition (See Ref [1],[2]. The authors describe in detail the process of diagnosis presenting appropriately labeled representative figures. However, it would be helpful for the readers if the histological images are presented with a scale bar of magnification.

 Overall, a well-presented case report to add to the existing knowledge on this congenital malformation.

Are enough details provided of any physical examination and diagnostic tests, treatment given and outcomes?

Yes

Is the case presented with sufficient detail to be useful for other practitioners?

Yes

Is sufficient discussion included of the importance of the findings and their relevance to future understanding of disease processes, diagnosis or treatment?

Partly

Is the background of the case’s history and progression described in sufficient detail?

Yes

Reviewer Expertise:

Cell biology and therapeutic strategies of Large /giant congenital nevi and neurocutaneous melanocytosis.

I confirm that I have read this submission and believe that I have an appropriate level of expertise to confirm that it is of an acceptable scientific standard.

## References

[1] : Do you know this syndrome? Schimmelpenning-Feuerstein-Mims syndrome. *Anais Brasileiros de Dermatologia* .2019;94(2) : 10.1590/abd1806-4841.20197661 227-229 10.1590/abd1806-4841.20197661 PMC648607531090832

[2] : Somatic KRAS mutation in an infant with linear nevus sebaceous syndrome associated with lymphatic malformations: A case report and literature review. *Medicine (Baltimore)* .2017;96(47) : 10.1097/MD.0000000000008016 e8016 10.1097/MD.0000000000008016 29381910 PMC5708909

# Response

**Published:** 2024-03-26

**Authors:** Kaveri Rusia

**Affiliations:** dermatology, datta meghe institute of higher education and research, wardha, maharashtra, India

Thank you for your response.
